# Agency From a Radical Embodied Standpoint: An Ecological-Enactive Proposal

**DOI:** 10.3389/fpsyg.2020.01319

**Published:** 2020-06-26

**Authors:** Miguel Segundo-Ortin

**Affiliations:** School of Liberal Arts, University of Wollongong, Wollongong, NSW, Australia

**Keywords:** agency, ecological psychology, enactivism, affordances, habits, sensorimotor schemes, information

## Abstract

Explaining agency is a significant challenge for those who are interested in the sciences of the mind, and non-representationalists are no exception to this. Even though both ecological psychologists and enactivists agree that agency is to be explained by focusing on the relation between the organism and the environment, they have approached it by focusing on different aspects of the organism-environment relation. In this paper, I offer a suggestion for a radical embodied account of agency that combines ecological psychology with recent trends in enactive cognitive science. According to this proposal, while enactivism focuses primarily on describing how our acquired sensorimotor schemes and habits mutually equilibrate, affecting our tendency to act upon some affordances instead of others, ecological psychology focuses on studying how perceptual information contributes to the actualization of the sensorimotor schemes and habits without mediating representations, inferences, and computations. The paper concludes by briefly exploring how this ecological-enactive theory of agency can account for how socio-cultural norms shape human agency.

## Introduction

Enactivism and ecological psychology are the two main schools of thought in the radical embodied cognitive sciences. They have much in common. Both approaches reject that cognition is confined to the head, and prompt for explanatory strategies that break away with the idea that cognition is based on the manipulation of mental representations. Likewise, both approaches stress the active role of the body and the environment in the processes that bring about cognitive activities.

In light of these affinities, a number of authors have already suggested the possibility of a unified approach to cognition that combines both theoretical frameworks (Chemero, 2009; McGann, 2014; Baggs and Chemero, 2018; Kiverstein and Rietveld, 2018; Heras-Escribano, 2019). Inspired by these proposals, this paper aims to contribute to this unification by focusing on the issue of agency. In a minimal sense, we can understand agency as the capacity of individual organisms or systems to execute goal-directed or intentional actions. So conceived, agency is manifested whenever an individual “acts on its own behalf in an environment” (Kauffman, 2000, p. 8), or when she does “something by itself according to certain goals” (Barandiaran et al., 2009, p. 369). In what follows, I will focus on this basic form of agency, and I will not discuss other forms, such as collective agency.

The dominant account of agency in philosophy – also referred to as “the standard theory” of agency (Schlosser, 2019) – assumes that a particular event *x* is an *action* just in case it has been appropriately (non-accidentally) caused by a series of mental states which represent both my goal and the actions intended to pursue that goal^1^. There are nonetheless reasons to think that the standard theory does not provide a satisfactory account of agency. For one thing, it has been pointed out that the standard theory is “too demanding,” for there might be organisms “that are capable of genuine agency and that do not possess representational mental states” (Schlosser, 2019). Besides, the fact that we currently lack a convincing story of how mental states can be causally relevant to behavior in virtue of their representational content undermines the credibility of the standard theory. These and other related problems have motivated defenders of 4E (embodied, embedded, extended, and enactive) theories of cognition to articulate alternatives to this standard account, emphasizing embodied and situated aspects of action (see, e.g., Juarrero, 1999; Malafouris, 2013).

Interestingly, even though both ecological psychologists and enactivists agree that agency must be explained by focusing on the relation between the organism and the environment instead of the individual’s representational states, they have evolved their own individual (and largely independent) approaches to it, focusing on different aspects of the organism-environment relation. Taking stock of this, the purpose of this paper is twofold. Firstly, I highlight the limitations of both the ecological and enactive approaches to agency, showing why they can’t offer a satisfactory account of it. Secondly, I show how ecological psychology and enactivism can complement each other to explain agency, overcoming their theoretical limitations. The complementarity approach I envisage can be summarized as follows: While enactivism focuses on investigating how the history of interactions of an organism gives rises to a series of sensorimotor schemes and habits that in turn play a causal role in determining how she interacts with the affordances of the environment, ecological psychology, through the notion of ecological information, explains how the individual can access to the environment’s affordances without mediating representations and inferences. It is argued that the existence of ecological perceptual information is essential to understand the organism-environment coupling that, according to enactivists, gives rise to action.

The structure of the paper is as follows. In section “The Issue of Agency in Ecological Psychology,” I analyze the most significant theories of agency proposed by ecological psychologists – Reed’s (1993,^1996^) theory of intentions and Withagen et al.’s (2012,^2017^) theory of affordances as invitations – and offer reasons as to why these proposals are unsatisfactory. Then, following the suggestion of Baggs and Chemero (2018) that ecological psychologists should adopt the theory of agency of enactivism, section “Enactive Agency” focuses on analyzing the enactive accounts of biological and sensorimotor agency. However, I argue that enactivism does not offer a satisfactory theory of agency either, for it leaves undetermined how the organism can access to the environmental structures that are relevant to agential behavior – an aspect that, we shall see, is essential to enactivism. Consequently, in section “Steps Toward the Unification”; I propose a dual approach to agency that combines the tools provided by enactivists and ecological psychologists. Nonetheless, it is well-known that the relation between ecological psychology and enactivism has not been an easy one. This could lead us to think that such a unified theory of agency is untenable. Section “Bridging the Uncanny Valley” confronts this view by addressing the most significant disagreements between enactivists and ecological psychologists. These disagreements pertain to, for instance, the notion of information at use in ecological psychology, or the nature of “sense-making” coined by enactivists. As I shall argue, most of the tensions between enactivists and ecological psychologists are based on reciprocal misinterpretations. To finish up, section “Conclusions and Directions for Future Research” concludes by briefly exploring the potential of this ecological-enactive approach to agency to explaining how human agency can be shaped by socio-cultural norms and conventions.

## The Issue of Agency in Ecological Psychology

The ecological theory of perception was first introduced by psychologist James J. Gibson in two seminal books published in Gibson (1966) and Gibson (1979/2015), respectively. The main ideas of this approach are: (i) that perception is direct; (ii) that perception is active; and (iii) that perception is action-oriented.

First of all, ecological psychologists are famous for arguing that perception is direct. To say that perception is direct is equal to saying that it is not mediated by representations. Instead, perception is conceived of as information detection. Perceptual information is given by higher-order properties of spatial-temporally extended patterns of stimulation – the so-called “invariants”^2^. According to ecological psychologists, since the invariants specify (lawfully correspond to) the properties of the environment that give rise to them, they provide non-ambiguous information about these environmental properties. Therefore, detecting these invariants is enough to be aware of the environment without the need for representations and inferences.

The second principle states that perception is active. Ecological psychologists reject the standard view of perception as a passive, sub-personal process that takes place inside the organism. Instead, perception is conceived of as “an achievement of the individual” (Gibson, 1979/2015, p. 228). The reason for this is twofold. On the one hand, the detection of information requires that the agent actively modulates its attention to detect the invariants that are relevant to their goal. In this sense, perception is not something that happens in the animal, but something the animal *does*. On the other hand, it is often the case that the information needed to carry on a particular perceptual task is not available in the array, but needs to be produced by the organism itself. To see this, think of motion parallax – the continuous and regular transformations of the apparent position of the objects from the starting point to the endpoint of the movement. Because the objects that are closer to the organism appear to move faster than those that are located further away, detecting the different speeds at which the objects “move” allows the organism to be aware of the different distances she holds regarding them. Moving as to produce motion parallax is thus an efficient strategy for perceiving depth.

Third perception is action-oriented. According to ecological psychologists, not only does action serve perception (as in the case of motion parallax), but perception is primarily for the control of the action. This idea is best captured by the claim that the main objects of perception are the affordances – this is, the opportunities for interaction that an environment (including other organisms) offers to an individual. Accordingly, when we detect information relative to the properties of an object (its rigidity, its size, etc.) we are primarily aware of its affordances – the possibility of grabbing it, throwing it, and so on. Hence, it is by directly detecting information that organisms can make their way in the world.

Some Gibsonians, however, have argued that the main explanatory target of ecological psychology is not perception, but agency. This idea is captured by Reed when he claims that “the goal of ecological psychology is to explain agency scientifically, not to explain it away or simply offer a discourse about it” (Reed, 1996, p. 19; see also Gibson, 1994). Three reasons justify the importance of agency for ecological psychologists (see Brancazio and Segundo-Ortin, 2020). Firstly, since perception requires the active and purposeful exploration of the environment by the animal, perception is already infused with our goals. Secondly, because affordances are opportunities for action, not causes of behavior, perception of affordances is not enough to regulate action. Rather, the organism must act upon these affordances (Gibson, 1967/1982, p. 411). Thirdly, because a single object offers multiple potential affordances to an animal, how she behaves is unconstrained by the affordances of the object. The animal must *select* what affordance to actualize at each moment (Cutting, 1982). Following this reasoning, I agree with Wagman (2019) that to adequately explain how perception contributes to action, we need a theory of agency, which is a theory of how individuals “select, perceive, and actualize affordances appropriately based on intention” (p. 148).

In what follows, I shall examine the two most significant attempts to explain agency from the ecological side: Reed’s (1993,^1996^) theory of intentions, and Withagen et al.’s (2012,^2017^) theory of affordances as invitations.

### Reed’s Theory of Intentions

At the beginning of his *Encountering the world*, Reed (1996) complains that “psychologists have persisted in modeling animal and human behavior on mechanical principles, thus neglecting perhaps the most fundamental problem of their field – autonomous agency” (p. 10). Reed (1996) characterizes agency as the capacity to put attention and action at the service of one’s current intentions (see also Gibson and Rader, 1979). As he explicates, “in any situation, an individual’s intentions serve to select a small number of the potential affordances available in that situation. This selection is reflected in the organization of the individual’s attention and activity” (Reed, 1993, p. 46). He nonetheless rejects the mainstream view of intentions as mental states that cause actions, and proposes the following characterization instead:

From an ecological point of view, intentions are not causes of action, but patterns of organization of action; they are not mental as opposed to physical, but are instead embodied in the kinds of performances most likely found in cognitively capable creatures. … The development of intention is thus the development of the ability to nest bouts of exploratory and performatory behavior so as to achieve desired outcomes (Reed, 1993, p. 62).

But, how do intentions emerge, if not in the mind of the perceiver? According to Reed, an intention can only emerge whenever there are multiple affordances available for the organism to choose. To explain how an organism selectively acts upon some affordances instead of others, Reed takes inspiration from Darwinian evolutionary biology. He hypothesizes that intentions, just like any other biological entity, emerge out of processes of variation and selection. For him, the minimal units of analysis for a theory of intentions are perception-action cycles (PACs). Each PAC is specific to a particular affordance or, to be more precise, to the information which specifies a particular affordance. Then, Reed suggests that in situations where the organism is offered multiple affordances, the PACs enter a sort of competition, and this competition results in goal-oriented or intentional behavior – namely, the actualization of an affordance. As he himself writes, drawing a clear parallelism with Darwin’s theory: “Intentions are thus the “species” that emerge out of competition among perceptual and action processes for utilizing affordances” (p. 65).

Reed’s account nonetheless suffers from significant shortcomings. To begin with, Reed provides no clue about the conditions under which the PACs compete. All we know is that intentions to actualize particular affordances emerge out of the competitions between PACs, but it is not clear what the selection pressures that define this competition are.

Reed (1996) is famous for sketching a selectionist approach in which affordances, conceived as environmental resources, exert selection pressures and give rise to species equipped with action systems –systems that are specialized in taking advantage of particular affordances. The same proposal can be rehearsed here to explain the emergence of intentions. Indeed, Reed (1996, p. 18) tells us that “the relative availability (or non-availability) of affordances create selection pressures on the behavior of individual organism.”

Reed’s selectionist approach is under attack by current neo-Gibsonians (see, e.g., Withagen and van Wermeskerken, 2010). The reason for this is that his account is markedly adaptationist, thus conflicting with the current trends in evolutionary biology. In this adaptationist view, environmental pressure and natural selection shape organisms’ evolution by favoring some genetic mutations over others, while organisms remain as passive receptacles of genes. This asymmetric approach to evolution is deemed too simplistic by current evolutionary biologists, who emphasize the active role of the organisms in altering the selection pressures to which they are exposed (see Lewontin, 1983; Laland et al., 2016)^3^. In addition, Turvey has recently argued that adaptationism is incompatible with ecological psychology, for it perpetuates the dualism of organism and environment (Turvey, 2018, p. 16).

Moreover, we can think of actions that are either irrelevant for our survival (e.g., nail-biting) or harmful (e.g., smoking), but that nonetheless belong to our common behavioral repertoire. This is a problem for Reed’s account, since if the PACs that give rise to these intentional actions are either irrelevant or detrimental (mal-adaptive) for the organism, then it is hard to see how we can make sense of some people’s tendencies to bite their nails or smoke from the adaptationist approach^4^.

Another possibility, also sketched by Reed (1993, 1996), is that social interaction contributes to the development of particular intentions. In this view, the competition between PACs is influenced by the actions of others who instruct and correct the behavior of the individuals, teaching them “*what* affordances can be utilized by *whom* and *when*” (Reed, 1993, p. 52, emphasis original). Although appealing, this second possibility is quite limited, for it only applies, as Reed himself acknowledges, to animals with complex societies and sociocultural norms. Surely, human agency is constrained by the social norms that rule within the communities we inhabit, but a theory of agency based *solely* on social norms cannot pay the entire bill, for there are non-human animals that are capable of agency and do not have social norms. These shortcomings have led other neo-Gibsonians to formulate alternative accounts of agency.

### Affordances as Invitations

Whereas Reed (1993, 1996) attempted to explain agency by drawing from Darwinian evolutionary biology, Withagen et al. (2012, 2017) try to do so by relying on contemporary phenomenology (Dreyfus and Kelly, 2007). Withagen et al. begin by rejecting the notion of intention put forth by Reed. Then, they argue that if we want to understand agency in ecological terms, we must think of affordances not just as opportunities for action, but as invitations to act as well:

If we recognize that affordances can also invite behavior, we are forced to a conception of agency that puts the animal-environment relation much more central. When actively exploring the environment, the agent is attracted or repelled by some of its affordances, and the ensuing behavior is partly the result of these invitations. This means that to understand how animals make their way in the world, the inviting character of affordances should be taken central (Withagen et al., 2012, p. 257).

According to this view, the environment does not simply offer a neutral manifold of possibilities for acting. Rather, some of its affordances can also invite us to act a certain way, “with us bodily responding to these callings” (Withagen et al., 2017, p. 12). Importantly, this is not to equate affordances with invitations. Instead, the hypothesis is that, under certain conditions, some affordances can invite action, whereby the invitational character of the affordances “depends on the agent-environment relation” (Withagen et al., 2012, p. 256)^5^.

If phenomenology is called upon to provide the theoretical inspiration for this proposal, Withagen et al. (2012, 2017) look at industrial design and architecture to provide real examples of affordances as invitations. For example, Norman (1988/2013) demonstrated years ago that the design of an object affects the individuals’ usage of it, making some affordances more easily perceivable and, consequently, more likely to be seized^6^.

Withagen et al. (2012) mention other organismal factors that can contribute to making affordances invitations. These factors include the action capabilities of the agent, the evolution of the species, the cultural background of the individual, and, finally, the history of interactions of the individual. As they explicate, “this inviting character of the affordances depends on the agent-environment relation, arguably in a multidimensional way … This suggests that whether an affordance invites is an animal-relative property of the environment as well” (p. 256).

Nonetheless, even if we can find the characterization of affordances as invitations suggestive and theoretically valuable^7^, the issue remains as to see how this characterization helps us to advance in our understanding of agency. Two main issues remain to be solved. First, invitations to act are not causes of behavior either. In fact, Withagen et al. go on to characterize agency as “the animal’s capacity to modulate the coupling strength with these affordances – the agent can influence to what extent each invitation influences him or her” (Withagen et al., 2017, p. 14). Therefore, thinking of affordances as invitations does not fully resolve the problem of how to explain agency, for we still have to make sense of the capability of the individuals to modulate their relation to the inviting affordances^8^. Second, and more importantly, this account presupposes agency instead of explaining it. To see this, consider the following passage:

An affordance can invite behavior if and only if an agent perceives it. If affordances are not perceived (or even have not been discovered) they do not have the potential to attract (or repel) the according behavior of the agent. Hence, a prerequisite for affordances to invite is an actually present observer that actively explores the affordances of its environment (Withagen et al., 2012, p. 257).

Whereas the affordances of the environment exist independently of being perceived by the individual, for an affordance to invite it, needs to be perceived. This means that for affordances to invite, we need an organism that is already capable of exploring its environment, actively focusing its attention in some informational patterns instead of others. This is, for affordances to invite, we already need an agent.

In conclusion, even though a theory of agency can incorporate invitations (namely, to make sense of actions that we perform in an unconscious or pre-reflective way), conceiving of affordances as invitations leaves untouched core aspects of the notion of agency. Motivated by this, some defenders of ecological psychology have come to propose that Gibsonians should look for a theory of agency in the other main school of thought in the radical embodied cognitive sciences: enactivism. To quote Baggs and Chemero (2018):

Ecological psychology focuses on the nature of the environment that animals perceive and act in; enactivism focuses on the organism as an agent. Combining the two would seem to provide a complete picture of cognition: an enactive story of agency, and an ecological story of the environment to which the agent is coupled (p. 2).

Unfortunately, Baggs and Chemero do not elaborate on this proposal, and do not explain how the enactive theory of agency can fit into the ecological picture. In section “Enactive Agency,” I shall analyze what enactivists have said about agency^9^. Can enactivism provide an account for how different individuals selectively perceive and act upon the affordances of the environment?

## Enactive Agency

One of the most important ideas presented in *The Embodied Mind* (Varela et al., 1991/2016) is that cognition arises out from the active and reciprocal coupling between the agent and its environment. The enactive framework emphasizes the role of the agent in enacting, or bringing about, their own cognitive life, but rejects that it needs to be done through representations and internal computational operations. Like Gibsonians, enactivists put agency at the center of their research program.

In an attempt to clarify what the notion of agency amounts to, Barandiaran et al. (2009) provide the following working definition. According to them, a system qualifies as an agent if it meets three necessary and jointly sufficient conditions. First, individuality: For a system to be an agent, there must be a distinction between the system and its environment. This is not to say that the system must be completely detached from the external world, but that it must be able to maintain itself as something distinguishable from the latter. Second, interactional asymmetry: Even if the environment can cause the individual to act a certain way on particular occasions, for an individual system to be an agent, it must be able to modulate the coupling with the environment, initiating some processes and resisting the tendencies exerted by the medium when needed. Finally, normativity: According to this condition, an agent must have intrinsic goals, and these goals provide a normative reference against which an action can be considered a success or a fail. This last condition is crucial, for it allows us to distinguish actions, properly speaking, from other bodily phenomena such as trembles or spasms.

According to Di Paolo et al. (2017, 2018), these three conditions provide the basic characterization for a notion of agency, and they can be used to investigate the different forms in which agency can be manifested: biological, sensorimotor, social, and linguistic. In what follows, I will focus on the biological and sensorimotor forms of agency, as I take them to be the most directly relevant to ecological psychology.

### Biological Agency

Arguably, the official story of enactivism begins with the notion of “autonomy.” This concept was first introduced by Varela (1979; see also Barandiaran, 2017) to capture the peculiar dynamics of living systems. In Varela’s view, living systems owe their existence to what he dubs “organizational closure.” A system is organizationally closed when it is composed of a number of internal dynamical processes such that (i) they recursively depend on each other (each process is simultaneously a causal enabling condition for, and an effect of other processes), and (ii) they constitute the system as a unity that is recognizable and identifiable against its medium. Autonomy is regarded by enactivists as a necessary and sufficient condition to speak of an individual system.

The most basic form of autonomy can be seen in autopoiesis. Autopoiesis, also referred to as “material self-production,” refers to the capacity of living systems to generate and maintain their own identity as something distinct from the environment. According to Weber and Varela (2002), an autopoietic system consists of:

A network of processes of production (synthesis and destruction) of components such that these components:

1. Continuously regenerate and realize the network that produces them, and
2. Constitute the system as a distinguishable unity in the domain in which they exist (p. 115).

According to the theory of autopoiesis, living beings preserve themselves in virtue of being organized as a network of metabolic processes that generate their own components. So conceived, autopoiesis differs from other dynamical processes because of its reflexivity: “the autopoietic system is organized so as to produce that very organization” (McGann, 2007, p. 469).

To say that autopoietic systems are autonomous is not to say that they are self-sufficient. Crucially, it is because all living beings need to interact with the world to preserve their autonomy that they develop an individual perspective of the environment. At the very least, a system must be able to distinguish those aspects of the environment that are valuable or meaningful to its autopoiesis. The appearance of this individual perspective from which features of the world are perceived in relation to the autonomous system’s viability is what enactivists call “sense-making.” This capacity for sense-making is, according to enactivists, what distinguishes cognitive from non-cognitive systems, and it is key to understanding biological agency.

Thompson (2007) illustrates this view through bacterial chemotaxis. Because the bacterium exploits sucrose as a source of nutrient, it is attracted to sugar concentration, whereas other chemicals are neutral or repulsive. Crucially, although sucrose is a real entity of the bacterium’s environment, its status or meaning as food is not. Rather, the latter is linked to the bacterium’s metabolic needs. This is the sense in which enactivists claim that the meaningful or cognitive environment is “brought forth” by the organism’s activity. As Thompson (2007) explicates, for the sense-maker, “the environment becomes a place of valence, of attraction and repulsion, approach or escape” (p. 158). Thus, according to the enactive picture, being a sense-maker implies being ready to selectively act upon the affordances of the environment that are relevant to maintain autonomy.

Nonetheless, Di Paolo (2005) argues that autopoiesis alone provides a very thin understanding of sense-making and agency. The reason for this is that autopoiesis provides an “all-or-nothing norm” (Di Paolo, 2005, p. 436), according to which environmental features are relevant only in relation to their *direct* impact on the system’s autopoiesis. Two undesired consequences follow from this. First, autopoiesis leaves no space for the possibility that organisms act to actively avoid or seek situations on the basis of physical encounters that are not inherently lethal/non-lethal. Think, for instance, about the footprint left by a prey. This environmental encounter is relevant for the organism, if only as a reliable proxy for future autopoietically relevant affordances (nutrition). However, because the footprint does not physically affect the organism’s metabolism, it is deemed meaningless for the point of view of the theory. Second, autopoiesis does not conceive of the possibility that organisms actively seek to improve the conditions for self-production, e.g., by swimming up the sugar gradient.

To overcome these limitations, Di Paolo proposes to combine autopoiesis with adaptivity. A system is deemed adaptive when it is able “to regulate itself with respect to the boundaries of its own viability” (p. 430). To do so, the system must implement a set of second-order processes^10^ that allow it to actively monitor internal and external perturbations, putting environmental encounters in relation to the whole spectrum of its viable states and thus recognizing in these encounters the tendencies that can lead to the loss or improvement of its autopoiesis. Adaptive systems are thus a subclass of autopoietic systems that can recognize environmental features as meaningful in virtue of their virtual consequences, thus perceiving “graded differences between otherwise equally viable states” (p. 437).

Autopoiesis and adaptivity are thus said to provide the bedrocks for a theory of agency at the biological level (Di Paolo et al., 2017, 2018; Heras-Escribano, 2019). Whereas autopoiesis (and, more generally, autonomy) provides the basic norms, or goals, for the system, e.g., to maintain its precarious individuality, adaptivity provides the means by which otherwise irrelevant environmental features are meaningfully identified, increasing the system’s sensitivity to the affordances of the environment and enabling it “to distinguish a situation as a risk or an opportunity, to tell the difference between good and better, bad and worse” (Di Paolo et al., 2018, p. 33). Therefore, an adaptive autopoietic system will be able not only to perceive the footprint as an opportunity to feed, but will also appreciate whether following the track of the prey is the best option given the circumstances. Investigating the species-specific mechanisms that give rise to this “graded perception” is a crucial step to understand how different organisms asymmetrically regulate their action in environments with multiple competing affordances (Reed, 1993)^11^.

Yet, in a sense, biological agency is very limited. Since the kind of norms that arise out from biological autonomy concern to our self-individuation only, if we were *just* biological agents, the only affordances of the world that we would care for would be those that are related to our survival. Anything that is not directly relevant to being alive would be non-significant to us. At the same time, we would always avoid taking any action that could entail a risk to our biological integrity (actions such as drinking alcohol, smoking, and so on). As both conclusions are obviously false, we can safely infer that, at least for human beings, biological agency cannot be the whole story.

### Sensorimotor Agency

Recently, Di Paolo et al. (2017) have attempted to complement the previous account with a theory of what they call “sensorimotor agency.” This kind of agency is present in sophisticated organisms capable of learning and acquiring new behavioral repertoires, and while it is enabled by biology, it is underdetermined by it.

The hypothesis that justifies the extension of agency beyond the biological realm is that we can find a form of autonomy at the level of perception and action. Two notions are crucial for this idea. The first one is “sensorimotor scheme.” These are organized, mutually adjusted sequences of sensorimotor coordination patterns that the individual deploys in carrying out a specific task and have been established as preferable in light of some normative framework, be it internal or external to the individual (namely, considerations of efficacy, timing, precision, and so on)^12^. To understand what sensorimotor schemes are, think, for example, of the activity of cooking a recipe. If the recipe requires that we chop the zucchini to a specific thickness, I will need to coordinate the movements of my hands in order to keep the appropriate distance between the knife and my fingers. However, this coordination is only possible on the basis of the continuous perceptual experience, and then requires the establishment of a task-oriented sensorimotor pattern. In normal conditions, this sensorimotor pattern will not be enacted in isolation. On the contrary, while I am chopping the zucchini, I shall pay attention to the onion that I put on the pan, either by looking directly at it or by smelling it, to prevent it from getting burned. Hence, the task of cooking a meal requires the enactment of multiple sensorimotor patterns. At the beginning, these patterns are not well integrated (my hand coordination is rather clumsy, I cannot identify when the onion in the pan is burning, etc.), but as long as I get proficient in cooking this meal, these patterns get intertwined in the form of a scheme: a task-related, mutually supporting relation of coherent sensorimotor patterns. Next time I cook this meal, I will have the disposition to execute (“enact”) the same sensorimotor patterns I successfully enacted in the past.

It is important to realize, however, that the execution of a scheme is not innocuous. Rather, when a particular scheme is executed, it has an effect on other schemes, either preventing them from occurring or increasing the likeliness they will be enacted. This leads us to the realization that sensorimotor schemes, just like individual coordination patterns, can be organized in “clusters,” or networks of mutually coherent and enabling sensorimotor schemes. Drawing a parallelism with autopoiesis, these networks of schemes are regarded as the “individuals” that give rise to sensorimotor agency: “the behavioral analog to biological agency is a network of precarious but interactively self-sustaining sensorimotor schemes, i.e. a *self-asserting sensorimotor repertoire*, whose adaptive regulation is directed at the preservation of internal coherence and consistency” (Buhrmann and Di Paolo, 2017, p. 219). According to this picture, a network of schemes constitutes a particular autonomous system that “is reasserted by every successful act and challenged by every breakdown” (Di Paolo, 2019, p. 15).

Thus, whereas the study of biological agency requires that we focus on understanding how metabolic and adaptive processes give rise to a selective engagement with the biologically relevant affordances of the environment, the study of sensorimotor agency requires that we focus on understanding how sensorimotor schemes intertwine with others, forming mutually consistent networks. The network of sensorimotor schemes an organism embodies determines how she deals with the world – her sensorimotor “style” or “identity.” As such, when we face the world, most of the time we are already “equipped with a rich repertoire of ready-made, highly organized [sensorimotor schemes]” (Di Paolo et al., 2017, p. 81), some of them widespread across the species, and others acquired in our previous history of interactions. These schemes condition what affordances we perceive and act upon, making some actions natural, while others feel awkward or unfamiliar. Therefore when considered at the sensorimotor level, the affordances of the environment become relevant not only because they contribute to our survival, but because they bestow “the stability and coherence of [our] sensorimotor repertoire” (p. 39).

The other important notion is “habit,” where habits are regarded as “self-sustaining precarious sensorimotor schemes” (Di Paolo et al., 2017, p. 144). A sensorimotor scheme is deemed precarious whenever the elements that support it depend for their structural stability on the regular enactment of the scheme. It means that “if the habitual scheme is not enacted with sufficient frequency, the structures supporting it starts to lose the properties that enable it. Eventually, the capability to enact the scheme degrades and disappears” (p. 144).

Enactivists oppose to the traditional reading of habits as rigid patterns of behavior that get automatically activated in the presence of the right environmental cues. As explained by Barandiaran and Di Paolo (2014), this conception spans from Descartes and Locke to modern behaviorism, and regards habits as units that result from the association of ideas or between stimulus and response, thus opposing habits to intelligent actions. By contrast, enactivists take the notion of habit from Phenomenology and Pragmatism, and picture them as behavioral routines that can be flexibly changed or customized if the context requires it. Indeed, pretty much in line with Dewey (1922), Egbert and Barandiaran (2014) go as far as to suggest that habits are essential to cognition, thus breaking the dichotomy between habitual and intelligent actions^13^.

But, what do habits have to do with sensorimotor agency? The key lies in the idea that habits are “self-sustaining.” As Di Paolo et al. (2017) explicate, this means that “a habit “calls” for its exercise and its exercise in turn reinforces its durability” (p. 144). According to this view, the fact that a particular sensorimotor scheme is habitual entails that it is more prompted to be enacted. As such, while different sensorimotor patterns can be as effective as others to reach a particular goal, some of them “are preferred because they are habitual and comfortable” (p. 143). Acquired sensorimotor habits thus guide the way we relate to the external world, normatively defining a set of viable actions (affordances) that can contribute to their preservation:

According to the enactive approach, habits are self-sustaining networks of bodily, neural, and interactional processes that become a source of [non-metabolic] normativity for an agent, in such a way that the preservation of her habitual identities guides much of her perception, thoughts, and behaviors (Ramírez-Vizcaya and Froese, 2019, p. 7).

Enactivists thus propose to investigate sensorimotor agency by studying how sensorimotor schemes and habits develop and relate to each other forming complex ecologies that in turn affect the way we interact with the world. In fact, following the enactive lead, Kiverstein and Rietveld (2018) understand habits as “interrelated states of action-readiness that coordinate to multiple relevant affordances” (p. 154). To complement this approach, enactivists have proposed a theory that attempts to explain how habits couple and mutually stabilize, as well as the conditions that determine how sensorimotor schemes become habits. Admittedly, the enactive theory of sensorimotor learning (Di Paolo et al., 2017; Di Paolo, 2019) is still to be further developed and tested upon, but it constitutes a significant milestone in the radical embodied cognitive sciences.

## Steps Toward the Unification

Now that we have a thorough picture of the enactive theory of agency, it is time to come back to the suggestion made by Baggs and Chemero (2018). Can enactivism *alone* provide ecological psychologists with a theory of agency? My answer to this question is no. The reason for this has to do with the way enactivists characterize sensorimotor schemes.

According to the enactivist characterization, sensorimotor schemes (and networks of these) are grounded in the complex dynamical arrangement of certain properties within the agent (namely, musculo-skeletal structures and neural networks) that in turn give rise to specific sensitivities and dispositions to act. Despite this characterization, Di Paolo et al. (2017) are clear that sensorimotor schemes are not something a body possesses. Rather, they claim that these are “modes in which structures in the agent and structures in the environment meet and mutually stabilize” and that they “constitutively involve both the organismic body and its environment” (p. 152). But if the environment is constitutive of the organism’s sensorimotor schemes, it follows that agency is not a property that belongs to the organism or a system, but “a property of a *relation* between that system as its surroundings” (p. 110).

This means that in order to explain agency and account for how sensorimotor schemes and habits unfold, selectively exploiting some affordances of the environment instead of others, we have to account for how the organism can access to the environmental structures that complement and stabilize her sensitivities and dispositions. Without this complementary story, the enactive theory of sensorimotor agency remains incomplete:

Having powers and sensitivities required for action, in other words, is only half of the story. The other half is access to suitable accompanying conditions surrounding the agent, which in our world-involving perspective must themselves be active and concrete and not merely formal (Di Paolo, 2019, p. 212).

My claim is that ecological psychology can provide enactivists with this complementary story and then that it can contribute to explaining agency. According to this idea, what enables the organism to access the environmental structures that are relevant to its goals is the existence of perceptual information. This information is given in the form of spatial-temporally extended patterns of stimulation that lawfully correspond or specify the environmental properties that are relevant for the system’s sensorimotor repertoire. This point is rather important because, according to the Gibsonian tradition, it is the existence of ecological perceptual information what makes possible the coupling between the individual and the affordances of the environment, and then the coupling of perception and action, without the necessity of performing inferences upon mental representations^14^. Thanks to this lawful correspondence, the organism can directly perceive the possibility of passing by an aperture if she detects the structured energy distribution this aperture generates. Remove the specific information, and you will be back to the old problem of having to explain how organisms can access the environment based on ambiguous and impoverished stimuli.

I therefore propose a dual approach to agency that combines the tools provided by enactivists and ecological psychologists. While enactivists focus primarily on describing how our acquired sensorimotor schemes and habits mutually equilibrate, affecting our tendency to act upon some affordances instead of others, ecological psychologists focus on studying how perceptual information contributes to the actualization of sensorimotor habits without mediating representations, inferences, and computations. Thus, we can replace the rather unspecific claim made by Di Paolo et al. (2017) for something more concrete: agency is a property of the relation between the organism and its environment, where this coupling is made possible by the existence of ecological perceptual information the organism can directly detect and exploit in guiding its action.

The contribution of ecological psychology to the enactive theory of agency can be seen in the following two examples. First, ecological psychology can bring to the enactive theory of agency a series of well-tested theoretical and empirical methods that allow us to identify what informational patterns need to be detected to enact a particular scheme, carrying out its associated task. For example, thanks to Lee (2009), we know that the information required to control braking is “time-to-contact,” and that this information is present in the optical looming pattern produced by the approaching obstacle. The crucial aspect here is that since the optical looming specifies the time remaining until driver and object collide, it provides unequivocal information to the driver about the actions she can perform: namely, whether braking is still possible, or she should prepare herself for an imminent collision. Experimental evidence shows that the same information is exploited to intercept moving targets (Fajen et al., 2008) and that it can be detected by dynamic touch as well (Cancar et al., 2013). The literature on time-to-contact shows that ecological psychology provides both a formal (mathematical) characterization of the informational variables required to successfully perform different tasks and a series of concrete examples of the sensory patterns where these variables are manifested, as Di Paolo (2019, p. 212) demands.

Moreover, this unified approach allows us to advance a new characterization of sensorimotor mastery. In this picture, sensorimotor mastery depends on embodying habitual sensorimotor schemes that integrate action patterns with the appropriate task-specific ecological information. Coming back to the example of cooking, sensorimotor mastery in this context requires that I coordinate my actions with the information of the environment, enacting action schemes that are well attuned to the information that is relevant to the task I am performing – namely, the olfactory information that specifies that the onion is burning in the pan. The process of coming to detect this task-relevant information is what Gibsonians call “education of attention” (Jacobs and Michaels, 2007). However, since the perceptual information needed to perform a task is not always present in the immediate environment, sensorimotor mastery also requires that we learn how to act in order to produce it. For example, the lion can learn that an efficient strategy to perceive whether the prey is reachable is to produce motion parallax, and can incorporate this act within its broader hunting-related sensorimotor schemes. Similar examples can be found in the literature about “dynamic touch,” where perceivers actively and skillfully manipulate objects in order to perceive their affordances (see Turvey and Carello, 2011).

In conclusion, rather than simply taking the enactive theory of sensorimotor agency from enactivism as Baggs and Chemero (2018) propose, I hold that ecological psychologists, through the notion of ecological perceptual information, can contribute to explaining it.

## Bridging the Uncanny Valley

In the previous section, I have proposed an approach to sensorimotor agency that combines enactivism and ecological psychology. However, it has been recurrently pointed out that there exist essential tensions between both research programs. Di Paolo et al. (2017) nicely captures this view when he claims that “the relation between the schools of thought [enactivism and ecological psychology] is one of strange familiarity, as if their respective practitioners were staring at each other across an uncanny valley” (p. 18, ff. 3). In what follows, I shall attempt to bridge this valley. To do so, I will address the most significant reasons that ground the tension between these schools of thought. My purpose is to show that these tensions are based on reciprocal misinterpretations.

To begin with, it is well-known that Varela et al. (1991/2016) conceived of enactivism in opposition not only to classical cognitivism, but also to ecological psychology. Whereas they agree with Gibsonians that perceptually guided action need not be explained by positing mental representations, they disagree with the explanatory strategy put forth by ecological psychologists. As they argue, the ecological picture gives no explanatory relevance to the organism’s own activity, and instead tries to explain perception entirely from the side of the environment:

For Gibson, these optical invariances, as well as the environmental properties they specify, do not depend in any way upon the perceptually guided activity of the animal (though Gibson’s followers do relativize them to a given animal niche). … In a nutshell, then, whereas Gibson claims that the environment is independent, we claim that it is enacted … Thus the resulting research strategies are also fundamentally different: Gibsonians treat perception in largely optical (albeit ecological) terms and so attempt to build up the theory of perception almost entirely from the environment. Our approach, however, proceeds by specifying the sensorimotor patterns that enable action to be perceptually guided, and so we build up the theory of perception from the structural coupling of the animal (p. 204).

Varela et al.’s diagnosis that ecological psychology neglects the structural coupling of organism and environment can also be found in the work of current enactivists. Consequently, they keep presenting enactivism as opposed to ecological psychology:

We agree with ecological psychologists when they highlight that real environments are rich enough to access directly their relevant meaningful aspects. We think they are in fact too rich, and that sense-making always involves a massive reduction of all the environmental energies that might affect the agent, to those within the dimensions of biological, sensorimotor, and social historically contingent meaning (Di Paolo et al., 2017, p. 227).

I hold that this position is based on a misreading of ecological psychology. For one thing, we must note that affordances – the primary objects of perception for ecological psychology – are organism-dependent. For example, that the glass I have in front of me is *graspable* is not a property of the glass alone, but a property that holds in virtue of the relation between the glass and myself. It is because I am equipped with hands of a certain size and opposable thumbs that the glass affords *graspability* to me, but it will not afford the same action to my cat. This is why J. J. Gibson always insisted that affordances point two ways, to the environment and to the observer (Gibson, 1979/2015, p. 121, 132). Moreover, the affordances do not depend on physical relations alone, but need to be related to the observer’s capabilities as well (Chemero, 2009). For example, studies have shown that the perception of the *climbability* of a step is susceptible to change as the perceiver ages or gets physically tired (Konczak et al., 1992).

Yet it is not only the affordances that imply the complementarity of animal and environment but the information too. At the beginning of *The Ecological Approach to Visual Perception*, Gibson (1979/2015) introduces a crucial distinction between the environment and the physical world. As he explicates, while the physical world comprises everything “from atom to galaxies” (p. 4), the environment refers only to those aspects of the world that can be detected and interacted with by a particular organism. Ecological information is said to be in the environment, not the world *per se*, meaning that the notion of information is relational as well (Segundo-Ortin et al., 2019). For example, whereas electromagnetic fields constitute information for sharks in the sense that sharks can detect and exploit them, they do not have the same status for human beings. Even though electromagnetic fields are real physical properties of the world, their status as perceptual information is determined at the ecological scale – the scale of the perceiver.

On the other hand, although Gibsonians put the emphasis on the environment when explaining perception-action, they do not claim that the environment alone suffices to cause it. By contrast, as we mentioned before, ecological psychologists conceive of perception as a sort of activity – something the animal does. It requires that the organism actively forages for information, sometimes moving in order to give rise to and perceive the required invariant patterns. This clearly shows that ecological psychologists do not obviate the role of the individual in bringing about or “enacting” its own perceptual world, as enactivists claim^15^.

Therefore, we can conclude that it is wrong to assert that ecological psychologists aim to explain perception only from the side of the environment. In fact, several Gibsonians have held that the correct unit of analysis for an ecological theory of perception is the organism-environment system, emphasizing the structural coupling, or “mutuality,” of both relata (see, e.g., Michaels and Carello, 1981; Richardson et al., 2008; Turvey, 2018).

Di Paolo et al. (2017) advance a complementary reason to feel unease about ecological psychology. According to them, even though ecological psychology rejects representationalism, it keeps committed a “functionalist general approach to cognition” (p. 18, ff. 3) that is incompatible with enactivism. For them, this is shown by the fact that ecological psychology explains perception in terms of information gathering, use, and transformation. The same idea has been coined by Hutto and Myin (2017, p. 86), for whom ecological psychologists’ use of “information pickup” reveals “an underlying commitment to an information processing story.”

Once again, I hold that this reading is misguided. Even though ecological psychologists use “information pickup” and “information detection” interchangeably, it does not follow that they hold that perception requires internalizing and processing information. Instead, an organism is said to pick up information whenever she tracks the dynamic patterns that are present in the topology of her sensory array, perceiving what this information affords. Reed makes this point clear when he claims that “ecological information cannot be transmitted: it is ambient and available, not something put over a channel; it is something to be detected or used (or not) in regulating action. … Information pick up is not a process of “internalizing” information” (Reed, 1996, p. 155). And the same idea is expressed by J. J. Gibson:

I do not believe that the visual system is a channel for transmitting signals from the retina to the brain. I believe it is a system for sampling the ambient array. … And that means that the observer’s brain cannot be compared to a computer, or to a processor of information delivered to it Gibson J. J. (1970/1982, p. 86).

Perception, in ecological psychology, does not consist of coding and passing messages from the sensory organs to the brain to be further decoded and computed, but on tracking properties in the sensory array and exploiting them to coordinate action *in situ.* Therefore, we can conclude that the reasons advanced by Di Paolo et al. (2017) to attack ecological psychology are misguided.

Yet enactivists are not the only ones to have expressed doubts upon the possibility of a unified framework. Attacks have come from the ecological side as well. For example, it is a common assumption among the Gibsonians that enactivists subscribe to a kind of mental constructivism that is radically incompatible with ecological psychology. Fultot et al. (2016) situate this problem in the way enactivist use the notion of “sense-making.” For them, because enactivists are “in favor of interpreting the activity of perceptual agents as a kind of construction of perceptually meaningful world” (p. 298), enactivism “is germane to the representationalist, not ecological, theory of cognition” (p. 304; see De Jesus, 2016; Hutto and Myin, 2017 for similar claims). Elaborating on the same issue, Heras-Escribano (2018) asserts that “if enactive agency emphasizes subjectivity, it cannot be compatible with ecological psychology” (Heras-Escribano, 2018, p. 136).

I nonetheless think that a more charitable reading of enactivism is possible. Consider, for example, the way Di Paolo et al. (2018) characterize sense-making:

*Sense-making* is the capacity of an autonomous system to adaptively regulate its operation and its relation to the environment depending on the virtual consequences for its own viability as a form of life. Being a sense-maker implies an ongoing (often imperfect and variable) tuning to the world and a readiness for action (p. 33, emphasis original).

As we can see, Di Paolo et al. steer clear of the constructivist interpretation of sense-making. On this view, sense-making does not consist of the creation of subjective meanings by the individual, but on the discrimination of what in the environment is relevant for its survival and potential actions. I take this reading to be totally unproblematic with ecological psychology, and perfectly compatible with the view of perception as the selective detection of information about affordances.

Admittedly, more work is needed to build up a general ecological-enactive approach of the sort Baggs and Chemero (2018; see also Heras-Escribano, 2019) seek. However, it seems clear that some of the most well-known reasons for the tension between ecological psychology and enactivism are based on misunderstandings. I hope that once these misinterpretations are clarified, the possibility of building an ecological-enactive theory of agency looks more plausible.

## Conclusion and Directions for Future Research

Explaining agency is a major challenge for those who are interested in the sciences of the mind, and non-representationalists are no exception to this. In this paper, I have examined how the two most important schools of thought in the non-representational cognitive sciences, ecological psychology and enactivism, address the issue of agency. I have proposed that there is a mutual fit between enactivism and ecological psychology, and that both theories can complement each other to explain sensorimotor agency. According to this view, the environment, as conceived of by ecological psychologists, contributes to the emergence of agential behavior by providing the organism with information about affordances, and agential behavior depends on the enactment of habitual patterns that integrate structures at the level of the organism with action-specific ecological information. Therefore, while enactivists explain how the history of interactions of an organism gives rise to a series of sensorimotor schemes and habits (a “sensorimotor repertoire”) that in turn play a causal role in shaping its current perception and action, ecological psychology helps us make sense of the environmental informational patterns that contribute to the emergence of agential behavior. Remarkably, this proposal is not in conflict with the idea that some affordances can be perceived as invitations in certain situations (Withagen et al., 2012, 2017), but rather contributes to explain how different individuals, by means of embodying different sensorimotor repertoires, regularly perceive and exploit certain affordances instead of others.

Besides, I argue that this ecological-enactive approach can provide a more comprehensive account of agency than the one previously provided by ecological psychologists alone. To see this, consider the problem of explaining how our individual agency can be modulated by socio-cultural norms. Gibsonians have largely noted that our relationship with the affordances of the environment does not only depend on our capability to detect information. Rather, this relation is often influenced by the social pressures and norms that rule within the communities we inhabit. As explained by Heras-Escribano: “Our social norms and conventions share their space with our individual perception of affordances, and sometimes our norms exert some pressure for not taking certain affordances given some social conventions” (Heras-Escribano, 2018, p. 175).

Gibsonians have nonetheless gone into great pain when trying to explain how social norms can influence perception-action in a way that is consistent with the core tenets of ecological psychology (see, e.g., Costall, 1995, 2012; Heft, 2007, 2017, 2018; Heras-Escribano, 2018; Rietveld and Kiverstein, 2014). I suggest that an ecological-enactive approach to sensorimotor agency can provide us with new theoretical resources to address this challenge. A hypothesis that is consistent with this approach is that individuals, by interacting and collaborating with peers, learn and acquire particular sensorimotor schemes and habits (see Adolph and Hoch, 2019). If these schemes and habits already encode (albeit implicitly) the social norms that are distinctive of their community, then we have a way to understand how these norms can have an influence on the affordances we perceive and act upon.

Whether or not the ecological-enactive theory of agency fully takes off is yet to be seen. If we aim to fully explain human agency, we must be shown whether an ecological-enactive theory of agency can explain complex phenomena such as group action (Marsh et al., 2009) or long-term planning (Brancazio and Segundo-Ortin, 2020). Nonetheless, it seems that a combined approach along the lines I have suggested here can be mutually beneficial and opens up new and promising lines of research. Let us keep exploring the possibilities of an ecological-enactive cognitive science.

## Author Contributions

The author confirms being the sole contributor of this work and has approved it for publication.

## Conflict of Interest

The author declares that the research was conducted in the absence of any commercial or financial relationships that could be construed as a potential conflict of interest.

## Footnotes

1 Exactly what counts as “appropriate causation” is a matter of discussion. However, this qualification aims to rule out cases of deviant causation (see Wu, 2016).

2 In the ecological literature, it is common to distinguish between structural and transformational invariants (see, e.g., Michaels and Carello, 1981). In short, whereas structural invariants refer to the properties of the sensory stimulation that remain unchanged, underlying other variations, transformational invariants are the “modes of change” of perceptual objects.

3 Even though this idea has been popularized by Lewontin, it is already present in the work of American Pragmatist John Dewey (see Crippen, 2016, p. 235).

4 Some evolutionary biologists have nonetheless suggested that there might be factors, other than bestowing adaptive advantages to a species, that cause a genotype to be favored by natural selection (see Dawkins, 1976). I thank a reviewer for the pointer.

5 Gibson is ambiguous regarding the notion of “invitation”: “The concept of affordance is derived from these concepts of valence, invitation and demand but with a crucial difference. The affordance of something does *not change* as the need of the observer changes. The perception of its affordances should not be confused with the temporary special attraction it may have” (Gibson, 1979/2015, p. 130, emphasis original).

6 For instance, Norman suggests that by changing the design of a door, namely, by adding an easily reachable flat bar instead of a knob, we can “indicate” the user that she needs to push the door to open it (Norman, 1988/2013, p. 10). A first-pass objection to this idea is that it is not clear how industrial design proves that affordances may also be invitations. In short, making some affordances more easily perceivable is not the same as making these affordances *invitations*.

7 Other authors have further expanded the connection between the notion of affordances-as-invitations and agency. For instance, Rietveld and Kiverstein (2014) include invitations as a crucial element of their Skilled Intentionality Framework, and assert that “[i]n acquiring a skill the individual becomes increasingly expert at responding adequately and appropriately to the actions a particular situation invites” (p. 334). In a similar vein, Crippen (2019) incorporates invitations to discussions in aesthetics, emotional perception, and action, suggesting that “[m]ysterious smiles and settings likewise can be understood in terms of affordances because both suggest something worthwhile is hidden, thereby inviting approach, exploration and deeper penetration” (p. 11). Some others strongly disagree with this view, however. For instance, Heras-Escribano argues that “the alleged invitational character of affordances adds nothing to our understanding of the epistemic and ontological aspects of affordances *per se*. On the contrary, this inviting character might make affordances even more obscure to understand” (Heras-Escribano, 2018, p. 111).

8 Bruineberg and Rietveld (2014) have proposed to explain this modulation by appealing to the Free Energy Principle in neurodynamics.

9 In what follows, I will focus exclusively on the enactive approach first developed by Varela et al. (1991/2016), and extended in the works of Thompson (2007) and Di Paolo et al. (2017, 2018), as it is the branch that most prominently has focused on the issue of agency.

10 I deem these processes “second-order” because they are not constitutive of the system’s autopoiesis.

11 Whereas the general notion of adaptivity is made operational through concepts from dynamical systems theory (“state trajectories,” “attractor landscape,” “viability set,” etc.), Di Paolo (2005, p. 440) offers suggestions about what the mechanisms that give rise to adaptivity in bacterial chemotaxis might look like.

12 The conditions under which different coordination patterns cohere are referred to as “sensorimotor norms.”

13 Habits are pivotal in the way Dewey understand human cognitive live. For example, he argues that “[r]eason pure of all influence from prior habits is a fiction” (Dewey, 1922, p. 31). Elaborating on this view, Crippen (2016, p. 247) suggests that the Deweynian notion of habit could be the basis of an enactive theory of morality.

14 As Withagen and Chemero write: “Direct perception is a coupling between the perceiver and the environment, via information in the array” (Withagen and Chemero, 2012, p. 532).

15 Based on this idea, Baggs and Chemero (2018) introduce a distinction between “habitat” and “umwelt.” Whereas “habitat” refers to the environment for a species, the umwelt refers to the environment for a particular behaving organism. According to them, this distinction helps fostering our understanding of the active role of the organism in perception (and cognition).
